# Introduction to LJPC issue 8.6

**DOI:** 10.1080/17571472.2016.1256044

**Published:** 2016-11-15

**Authors:** Paul Thomas

**Affiliations:** ^a^ LJPC Editor

This issue of LJPC focuses on ethics in multidisciplinary and complex contexts.

(1) Papanikitas discusses how to use health care conferences to combine the study of ethics with efforts for ethical change. He concludes a need for workshops at conferences coupled with a facility to continue conversations in between conferences. He followed his own advice by leading a LJPC workshop at the 2016 RCGP conference that debated the need for both survival and flourishing of general practice – a theme to be continued in LJPC in 2017 when considering new models of care.
(2) Wiles et al. present a partially fictional case of a mother with HIV to illustrate how multidisciplinary groups can learn about ethical issues through reflection on their experiences and debate. What would you do if you came across a mother who was HIV positive?
(3) Clark presents an evaluation of the USHAPE (Ugandan Sexual Health and Pastoral Education) initiative to improve family planning services in Uganda. Their initial survey revealed many cultural, religious, social and practical barriers to women accessing family planning mean, even though half had an unmet need for it. By engaging local leaders, they were able to train 68 members of staff at a local hospital to a basic level, and a further 32 members of staff to the higher Level Two.

In 2017, LJPC wishes to publish more about ethical issues in multidisciplinary and complex contexts. Keep the papers coming. In the meantime, we wish all readers a happy, festive season.

Paul Thomas LJPC Editor

